# Interrogating the Essential Bacterial Cell Division Protein FtsQ with Fragments Using Target Immobilized NMR Screening (TINS)

**DOI:** 10.3390/ijms20153684

**Published:** 2019-07-27

**Authors:** Marjolein Glas, Eiso AB, Johan Hollander, Gregg Siegal, Joen Luirink, Iwan de Esch

**Affiliations:** 1 Division of Molecular Microbiology, Amsterdam Institute for Molecules, Medicines and Systems (AIMMS),Vrije Universiteit Amsterdam, 1081 HV Amsterdam, The Netherlands; 2 Division of Medicinal Chemistry, Amsterdam Institute for Molecules, Medicines and Systems (AIMMS),Vrije Universiteit Amsterdam, 1081 HV Amsterdam, The Netherlands; 3 Zobio BV, 2333 CH Leiden, The Netherlands

**Keywords:** bacterial cell division, antibacterials, *Escherichia coli*, fragment screening, divisome, FtsQ, NMR, TINS

## Abstract

The divisome is a large protein complex that regulates bacterial cell division and therefore represents an attractive target for novel antibacterial drugs. In this study, we report on the ligandability of FtsQ, which is considered a key component of the divisome. For this, the soluble periplasmic domain of *Escherichia coli* FtsQ was immobilized and used to screen a library of 1501 low molecular weight (< 300 Da), synthetic compounds for those that interact with the protein. A primary screen was performed using target immobilized NMR screening (TINS) and yielded 72 hits. Subsequently, these hits were validated in an orthogonal assay. At first, we aimed to do this using surface plasmon resonance (SPR), but the lack of positive control hampered optimization of the experiment. Alternatively, a two-dimensional heteronuclear single quantum coherence (HSQC) NMR spectrum of FtsQ was obtained and used to validate these hits by chemical shift perturbation (CSP) experiments. This resulted in the identification of three fragments with weak affinity for the periplasmic domain of FtsQ, arguing that the ligandability of FtsQ is low. While this indicates that developing high affinity ligands for FtsQ is far from straightforward, the identified hit fragments can help to further interrogate FtsQ interactions.

## 1. Introduction

Bacterial cell division is an essential and delicate process, and has therefore been suggested as a potential target for antibacterial treatment [1,2]. Cell division in Gram-negative bacteria involves a large protein complex called the divisome that mediates invagination of the inner membrane, synthesis of the cell wall, and inward growth of the outer membrane during the final stage of bacterial cell division. At least ten proteins of the divisome are indispensable for cell division, and the potential of these bacterial cell division proteins as promising antibiotic targets has been suggested several times [1,2,3]. To date, only the early cell division proteins FtsZ (a structural homologue of tubulin) [4,5] and ZipA have been studied in small molecule drug research [6,7].

Another divisome protein that has potential as a drug target is FtsQ, one of the late cell division proteins. FtsQ is an interesting target for several reasons. It plays a central role in the assembly of the divisome judged from the many different divisome proteins with which it interacts [8,9]. Furthermore, FtsQ has a very low cellular concentration of approximately 300 molecules per cell [10]. The protein is highly conserved amongst Gram-negative bacteria [11,12] and has no known human homologues. FtsQ forms a stable trimeric complex with FtsB and FtsL [13,14], the formation of which is essential for successful cell division. The interactions with FtsB and FtsL mainly take place in the periplasmic domains of these proteins, implying that drugs targeting these interactions would only have to cross the outer membrane of the bacterium. The interactions of FtsQ with FtsB and FtsL hence form an attractive target for intervention with small-molecule inhibitors, which could lead to the development of a novel class of antibiotics targeting bacterial cell division.

Protein-protein interactions (PPIs) have emerged as a highly relevant, but mostly untapped source of drug targets. Protein-protein interfaces are often large and shallow surfaces (approximately 500–1500 Å^2^ per side) [15], containing few features that offer productive binding sites for intervention with a small molecule. However, in the past decades, numerous small molecules targeting PPIs have been reported and studied in clinical trials [16]. The field of PPI modulators offers an enormous potential for the design of drugs against targets that have not been explored to date. Although the interface can be very large, the interaction energy of PPIs is usually not equally distributed over the whole interface but focused in hot spots: smaller regions within the interface that account for relatively high interaction energies [17,18]. These hot spots form promising targets for small-molecules that interfere with PPIs. Earlier, we have mapped the interactions of FtsQ with FtsB and FtsL biochemically using site-specific photo crosslinking. Interaction with FtsL was focused in the so-called POTRA domain whereas a hotspot for FtsB interaction was detected in the C-terminal domain of FtsQ near residue S250 [19]. Targeting these regions might be a potent strategy to interfere with FtsQBL complex formation.

Since the structure of the FtsQBL complex is not known, thereby precluding structure-based drug design, we decided to initiate this project by selecting compounds that interact with FtsQ in general. The rationale of this unbiased approach is that any compound that binds to the relatively small periplasmic domain of FtsQ could potentially inhibit, either directly or allosterically, the interaction with FtsB, FtsL or any of the other divisome proteins with which it interacts. To assess the ligandability of FtsQ and enable the development of molecules that bind to FtsQ, we followed an FBDD (fragment-based drug discovery) approach. Different from high throughput screening (HTS) methods, FBDD fragments with a low molecular weight (typically 150–300 Da) are screened. Fragments can be described by the rule of three (MW < 300 Da, cLogP < 3, H-bond donors < 3, H-bond acceptors < 3, number of rotatable bonds < 3 and TPSA (total polar surface area) < 60 Å^2^) [20]. The principle of FBDD is to develop drug leads by first identifying smaller components (fragment hits) that show molecular recognition at a given target [21]. The binding site of a target can be explored more efficiently with fragments, since these low molecular weight compounds cover chemical space more efficiently than larger drug-like molecules (300–500 Da). Therefore, fragment libraries are considerably smaller than high throughput screening (HTS) libraries. Furthermore, it appears easier to retain the good physicochemical properties of a fragment during the optimization process. Since fragments are so small, they have fewer molecular features than drug-like compounds, resulting in a higher probability of binding to a protein target but also resulting in low affinity hits (KD > 10 µM) [22].

In order to identify weak binding fragments, sensitive biophysical techniques like NMR, SPR or X-ray crystallography are required for detection [23], especially in the case of protein-protein interaction targets. Specifically, target immobilized NMR screening (TINS) [24] was used to find fragments that bind to the periplasmic domain of FtsQ. In this method, the target protein and a reference protein are immobilized in separate compartments of a dual-cell sample holder [25]. Mixtures of fragments are simultaneously injected into the target cell and reference cell, and the 1D ^1^H NMR spectrum for each mixture is recorded. Upon binding of a fragment to the target protein, the peak amplitudes of the fragment decrease relative to those in the reference cell. The comparison to the spectrum in the reference cell compensates for the effect of non-specific binding of fragments to protein surfaces or the resin. In order to facilitate the experiments, we used the soluble periplasmic domain of the transmembrane protein FtsQ (FtsQ_p_), which we have described in our previous work [13]. The obtained hits were validated using SPR and protein observed ^1^H,^15^N-HSQC NMR as orthogonal experiments.

## 2. Results

### 2.1. Site-Specific Immobilization of FtsQ_p_

To use FtsQ in TINS, an Avi-tag was introduced at the N-terminus of its soluble periplasmic domain allowing site-specific biotinylation. The functionality of the immobilized FtsQ_p_ was assessed by performing a pull down of its partner protein FtsB_p_L_p_ from solution. The immobilized protein was still able to form a complex with FtsB_p_L_p_ and thereby appeared to be functional. By this criterion, FtsQ_p_ appeared similarly functional after storage of the immobilized protein sample at 4 °C for 1 week. As a reference sample, the Pleckstrin-homology domain of Akt (Akt-PH) [26] was chemically biotinylated and immobilized on the resin in a similar manner.

### 2.2. Fragment Screening Using Target Immobilized NMR Screening (TINS)

**Figure 1:**
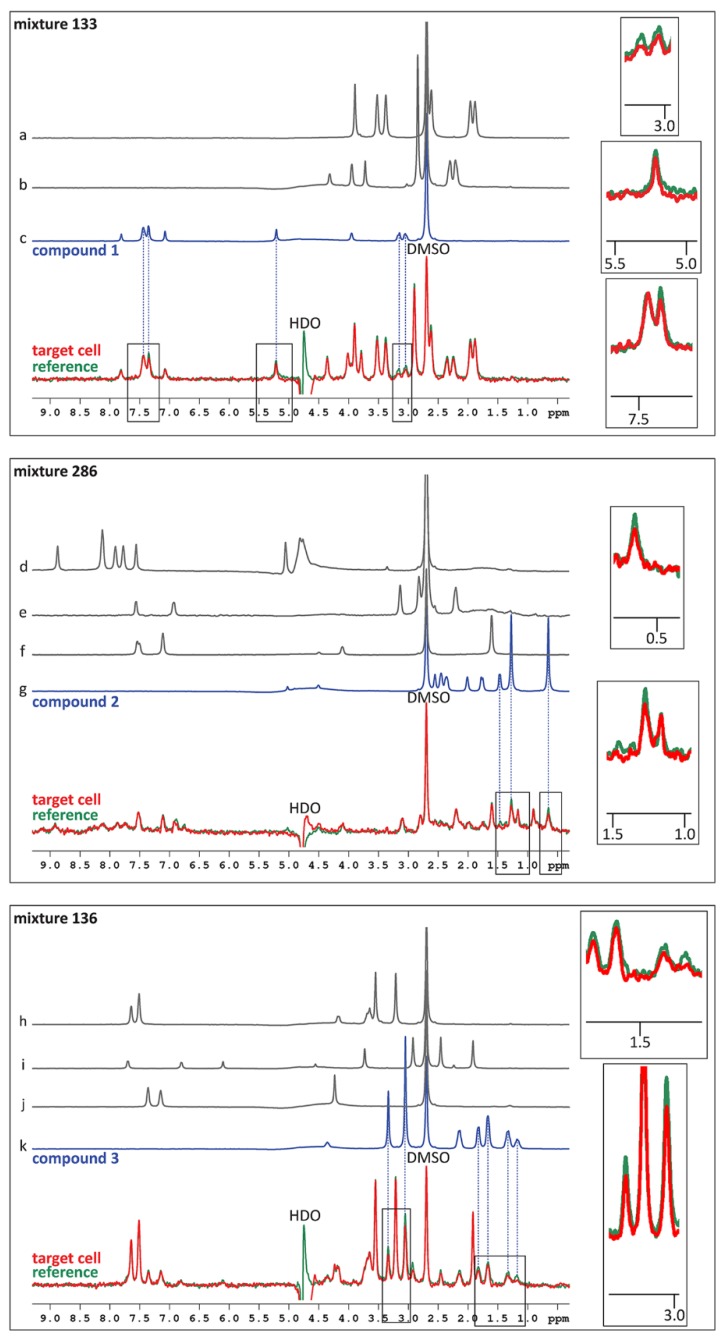
TINS spectra showing the mixtures containing compounds 1, 2 and 3. The blue traces show the ^1^H-NMR spectra of the compounds, the grey traces show the ^1^H-NMR spectra of the other compounds present in the mixtures. None of these other compounds were identified as a hit, as they gave T/R ratios between 0.930–1.045. Compounds 1, 2 and 3 resulted in T/R ratios of 0.839, 0.789 and 0.806, respectively. The red trace represents the spectrum obtained from the target cell, the green trace shows the spectrum obtained from the reference cell. The background signals caused by the solvent are annotated with DMSO and HDO. Hits were identified by calculating the ratio of the red (target) spectrum compared to the green (reference) spectrum: interaction with FtsQ_p_ causes a decrease in peak height in the target cell compared to the reference cell.

**Figure 2:**
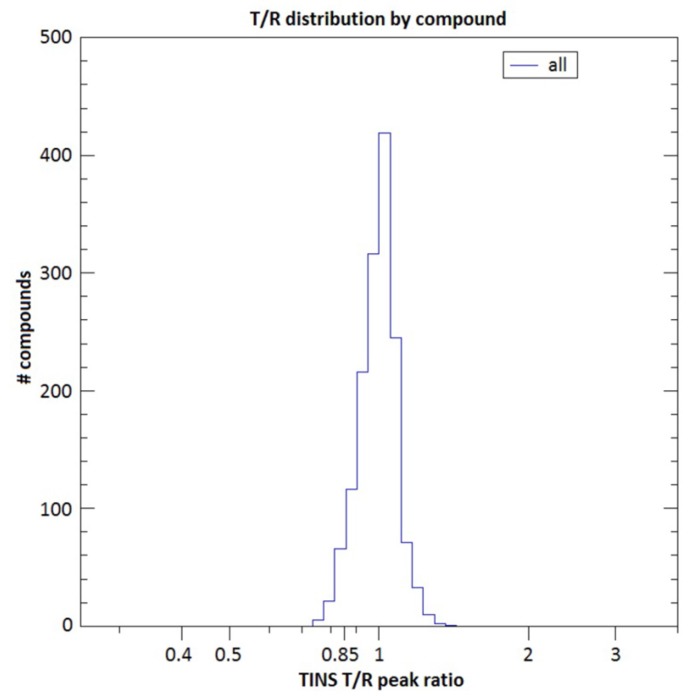
TINS profile of the screening. The TINS profile shows the compounds from the ZoBio fragment library binding to immobilized FtsQ_p_ (target) vs. Akt-PH (reference).

**Figure 3:**
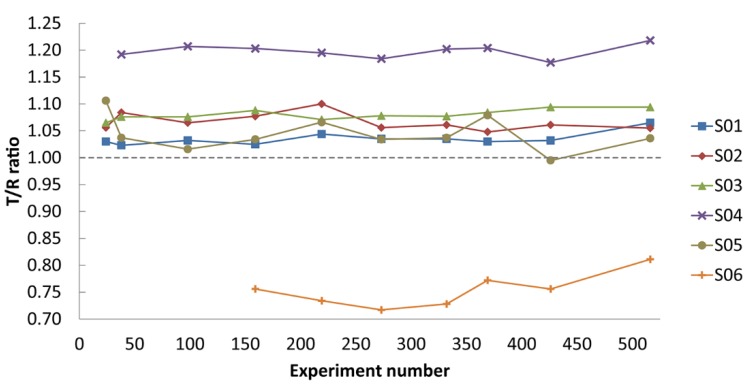
Stability of the screening. A control mixture of five or six compounds was repeatedly injected during the full course of the TINS screening (516 experiments) to monitor the stability of the immobilized proteins. The T/R ratio of these fragments showed no significant increase or decrease over time, from which we conclude that the protein in the sample holder was stable and properly folded during the entire experiment.

**Figure 4:**
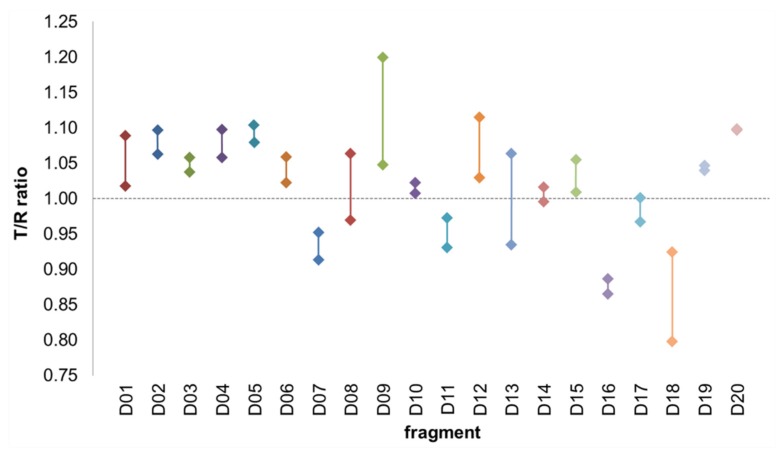
Sensitivity and reproducibility. As quality control, twenty fragments were run in duplicate. The median difference between two measurements was 0.04 and the average difference was 0.05. This shows that the sensitivity of the method is high enough to justify the chosen cutoff of T/R ≤ 0.85. Sixteen out of these twenty fragments had the same outcome, favoring either FtsQ_p_ (T/R < 1.00) or the reference protein (T/R > 1.00) in both experiments. Two fragments had a different outcome but a very small mutual difference < 0.05 close to T/R = 1 (D14 and D17), and two fragments did have a different outcome due to a mutual difference > 0.05 (D13 and D08).

In order to identify ligands for FtsQ, the ZoBio fragment collection (1501 fragments) [23] was screened for compounds binding to FtsQ_p_ at 500 µM each, in approximately 450 mixtures. All of the fragments included in this library obey the ‘rule of three’ [20]. The compounds have been selected according to four themes that include diversity using the scaffold-based classification approach, amino acid derivatives, scaffolds found in natural products, and shape diversity [23]. A spatially selective Hadamard NMR experiment [27] was used to simultaneously acquire a one-dimensional ^1^H spectrum of compounds in the presence of FtsQ_p_ and Akt-PH. The data resulting from the screen were analyzed without deconvolution because fragments could be directly identified by comparing peaks from TINS spectra with reference spectra of the individual fragments (Figure 1). The height weighted ratio of NMR resonances in the target and reference spectra (T/R ratio) from each fragment were determined post screen. The T/R ratios for all fragments were then bucketed and the resulting histogram provides a profile of the complete screen (Figure 2). Based on the large jump in the number of compounds in the T/R ≤ 0.85 bucket vs. the adjacent bucket of T/R >0.85, we chose 0.85 as a cutoff for hit selection. Thus, all compounds with T/R ≤ 0.85 were deemed to be preferential binders of FtsQ_p_ resulting in 72 fragment hits. However, the narrow T/R profile in Figure 2 is a clear indication of the poor ligandability of FtsQ. The selection of the T/R ≤ 0.85 was a result of the poor ligandability and desire to further investigate a significant number of potential ligands as more typically a cutoff of 0.7-0.75 is used. The spectra of the mixes containing the three hit fragments that were ultimately orthogonally validated (see below) are shown in Figure 1. The resulting hit rate for FtsQ_p_ was 4.8%, which is in the range typically observed using TINS (3%–10%) [28]. During the experiment, a control fragment mixture was repeatedly injected to confirm proper folding of the protein (Figure 3). The consistency of the measurements was confirmed by running 20 fragments in duplicate (Figure 4).

### 2.3. Hit Validation

After obtaining the 72 hits from the TINS screen, we aimed to validate the hits by an orthogonal assay and tested the compounds for affinity for FtsQ_p_ by SPR. In addition to the 72 hits, 120 structural analogues of the hit compounds were selected from our in-house libraries and tested in the SPR assay. Although a small-molecule control was not available at the start of the experiments, we aimed to obtain a control compound from the set of fragments itself, which then could be used to fully optimize the assay. At first, all fragments were tested in duplo at a single concentration of 750 µM. The six compounds that gave the highest response in this screen were titrated at concentrations ranging from 1 µM to 750 µM. However, even at the highest compound concentration, saturation could not be reached for any of these fragments, most likely because of the low affinity of the hits. The lack of a potent reference compound also hampered further optimization of the SPR assay. For this reason and because of its ability to show specific localized effects that in principle allow localization of the binding site, we decided to use ^1^H,^15^N-HSQC (heteronuclear single quantum coherence) NMR in addition to SPR to validate the TINS hits by identifying chemical shift perturbations (CSPs). Because of the limited throughput of the ^1^H,^15^N-HSQC experiment, twenty fragments were selected for further analysis.

**Figure 5:**
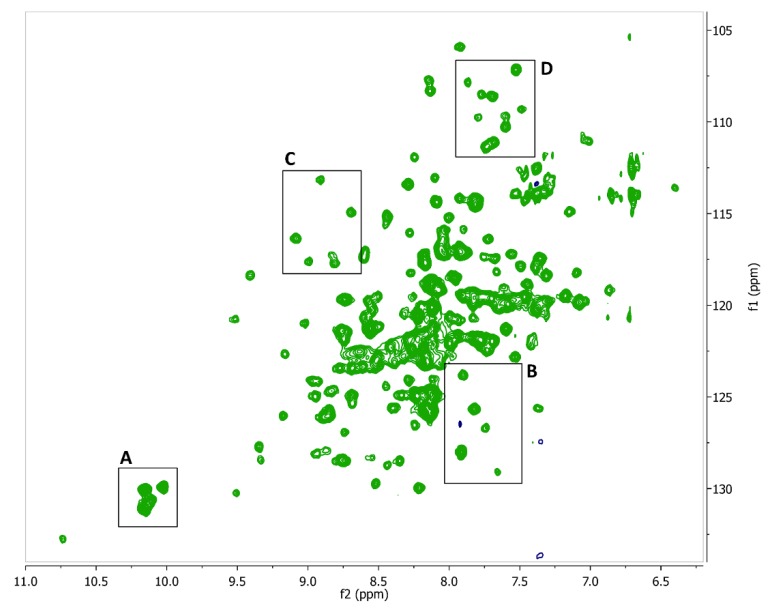
^1^H-^15^N HSQC spectrum of the periplasmic domain of FtsQ. The five tryptophan residues give clear distinguishable signals at a 10–11 ppm shift. Area’s (**A**–**D**) show the portions of the spectrum from which the zooms showing chemical shifts perturbations were taken, as shown in Figure 6.

**Figure 6:**
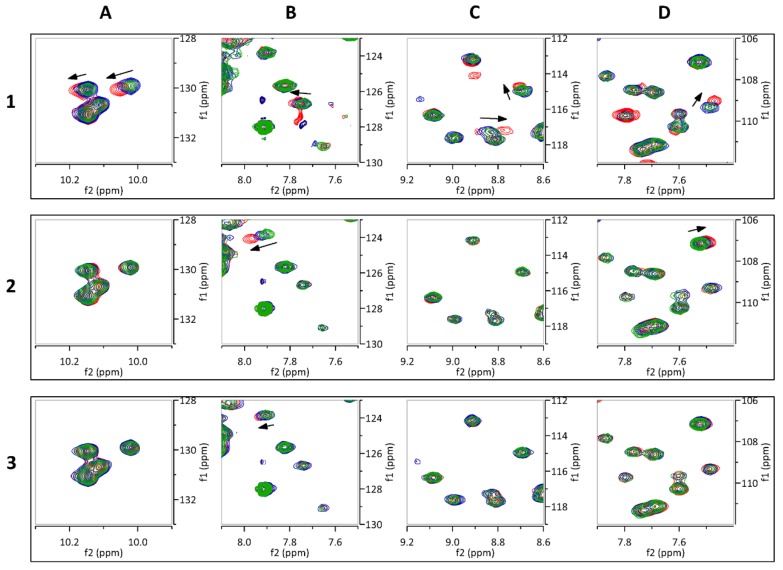
Chemical shift perturbations of FtsQ_p_ with compounds 1, 2 and 3. Each panel shows the superimposed spectra of 200 µM FtsQ_p_ without the fragment (green) and in solution with the fragment at 1 mM (blue), and 4 mM (red). Chemical shift perturbations are marked by black arrows. The areas of the close-ups shown in panels (**A**–**D**) are designated in Figure 5.

**Figure 7:**
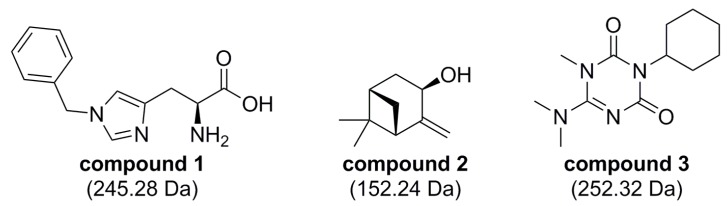
Structures of compounds 1, 2 and 3. The three identified hits are structurally different molecules. As they are fragments, these compounds all have a molecular weight below 300 Da.

Isotopically (^15^N) labeled FtsQ_p_ was expressed and purified in high yield (approximately 10 mg purified protein per liter culture). The obtained ^1^H,^15^N-HSQC NMR spectrum of FtsQ_p_ was indicative of a well folded, monomeric protein (Figure 5). The binding of each of the twenty fragment hits to FtsQ_p_ was assessed at two different concentrations by identification of CSPs. Three fragments were found to induce specific CSPs in the spectrum of FtsQ_p_ (Figure 6; Figure 7). The binding affinity of these fragments could not be determined in these experiments, as saturation was not yet reached at the maximum ligand concentration of 8 mM. Higher ligand concentrations were not possible due to limitations of solubility of the compounds. Analysis of the observed CSPs shows that the shifts caused by compounds 2 and 3 partially overlap, while the shifts caused by compound 1 are very different (Figure 6). These different CSP patterns suggest that compounds 2 and 3 bind to FtsQ_p_ at different binding sites compared to compound 1.

These results show that the ligandability of FtsQ is low, illustrated by the low number of hits that result from our screening that also prove to be of low affinity. Nonetheless, these results demonstrate the power of NMR as a sensitive screening technology. Although the affinity of the few compounds identified seems to be very low (> 1 mM), they might be valuable tools to interrogate FtsQ and as starting points for the development of higher affinity ligands for FtsQ.

## 3. Discussion

The need for novel classes of antibiotics is eminent, as all current classes of antibiotics are under the pressure of bacterial resistance. The bacterial cell division protein FtsQ has been proposed as a potential novel antibacterial drug target, as it is essential for bacterial proliferation and highly conserved amongst Gram-negative bacteria [1]. To date, no drug-like molecules have been reported to interact with FtsQ, nor with its binding partners FtsB or FtsL. In this work, we report the first screen of a compound library for binding to FtsQ. In order to identify such small molecules, we have chosen to use a fragment-based approach. In fragment-based drug discovery, libraries containing molecules of 300 Da or smaller are being screened. In contrast to conventional drug-like molecules of 300–500 Da size, typically screened in high throughput assays, fragments by being smaller are better able to cover their respective chemical space [29] and typical fragment libraries consist of 1000–1500 compounds. On the other hand, the small fragments generally have a lower affinity towards the target protein. Therefore, sensitive biophysical methods are needed to demonstrate binding rather than activity. We have used two different NMR approaches to identify and confirm fragments binding to FtsQ.

First, we used TINS to screen the fragment library for initial hit molecules, followed by SPR to make a selection of the hit fragments for further validation. Second, we performed protein-observed HSQC experiments to confirm interaction of the selected ligands with FtsQ. To enable experiments with FtsQ in solution, we used the truncated periplasmic domains of FtsQ, FtsB and FtsL, without their transmembrane segments. In our previous work, we showed that these soluble mutants (FtsQ_p_, FtsB_p_ and FtsL_p_) form a trimeric complex in vivo, and that the interactions in this soluble FtsQ_p_B_p_L_p_ complex are consistent with interactions in the native membrane embedded FtsQBL complex [13,19]. In earlier FtsQ_p_ labeling experiments, we found that chemical biotinylation of FtsQ_p_ inhibited its capacity to interact with FtsB_p_ (unpublished data). We therefore chose to introduce an Avi-tag at the N-terminus of FtsQ_p_ to site-specifically biotinylate the protein using BirA biotin ligase. Consequently, the orientation of FtsQ_p_ on the resin resembles the natural orientation of the periplasmic domain protruding from the inner membrane. After immobilization on the resin, a small-scale pull-down experiment was performed to prove that FtsQ_p_ was still capable to bind FtsB_p_L_p_, even after storing the immobilized protein at 4 °C for 5 days. From this we concluded that the protein was properly folded and stable for the duration of the complete TINS experiment. This was confirmed during the experiment by monitoring the consistency of output upon repeated injections of control compound mixtures during the course of the experiment (Figure 3). As a reference, we used the Pleckstrin-homology domain of the Akt kinase (Akt-PH), which is selected for minimal specific small molecule binding [26]. Because the entire fragment collection (1501 compounds) is screened using a single protein sample, a very small amount of protein is required (typically only ∼25 nmol), which makes TINS accessible for many targets. The ZoBio fragment library of 1501 fragments was screened using TINS with a single sample of less than 2 mg of protein.

Using a cut-off of T/R ≤ 0.850, the experiment resulted in 72 ligands that bind preferentially to the periplasmic domain of FtsQ. We accounted for non-specific binding by detecting the TINS effect caused by binding to FtsQ_p_ relative to Akt-PH that was immobilized under identical conditions. It should be noticed that the profile of the TINS screen was very narrow (Figure 2), suggesting that the ligandability of FtsQ is low. The minimum T/R ratio measured in the TINS screening was 0.739. This might be explained by the fact that the periplasmic domain of FtsQ mainly displays shallow surfaces, and does not contain very deep cavities or distinct binding pockets.

It was intended to use SPR to validate these hits, but due to the lack of a potent positive control compound we were not able to determine the affinity or kinetics of the hit fragments by SPR. Since the ligand binding was extremely weak and no positive control was available, it was not possible to fully optimize the SPR assay and obtain reliable data. However, as some of the fragments did give a response on SPR while others gave no response at all, these results were used to narrow down the hits from the TINS screen to a smaller number of compounds for further investigation.

The selection of TINS hits was validated by CSP experiments using the ^1^H,^15^N-HSQC spectrum of FtsQ_p_, which resulted in the identification of three confirmed fragment binders (compounds 1, 2 and 3). Comparing the CSPs caused by these three fragments shows that the CSPs of compounds 2 and 3 largely overlap, while a clearly different pattern is observed for compound 1. This suggests that compound 1 occupies a different binding site than compounds 2 and 3. Compound 1 causes a large shift of one of the five tryptophan residues (Figure 6A). Since the periplasmic domain of FtsQ contains only five tryptophan residues, the binding site of compound 1 could be revealed by assigning the five tryptophan signals in the spectrum.

During preparation of this manuscript, a partial structure of FtsB in complex with the periplasmic domain of FtsQ was resolved that reveals an interaction of residues 64–87 of FtsB with the C-terminal end of FtsQ [30]. Although this could be the basis for an alternative structure-based approach to identify inhibitors of this interaction it should be noted that FtsL is not present in this assembly, which might influence the conformation of the interaction interface.

Since the resonance assignment of the ^1^H,^15^N-HSQC NMR spectrum of FtsQ_p_ is not yet available, it is difficult to predict the binding site of the three hit fragments. Clearly, this would be the next step in further investigation of the identified compounds. Obtaining the assignment would likely reveal the approximate binding sites of each of the validated fragment hits. This detailed information would aid in the design of more potent compounds. When higher affinity compounds would be identified, these second generation compounds would be subject to functional assays like competitive NMR or SPR experiments.

It should be noted that, unlike compounds 2 and 3, compound 1 causes a clear shift of one of the tryptophan residues of FtsQ_p_ found at 10.0–10.2 ppm. Since the periplasmic domain of FtsQ only contains five tryptophan residues, this finding considerably narrows down the number of possible binding sites for compound 1.

In summary, this is the first attempt to find small-molecule ligands for FtsQ. Although FtsQ was found to be a challenging drug target, three weak affinity ligands were identified using TINS as a tool for fragment screening, showing the sensitivity of the experimental approach.

## 4. Materials and Methods

### 4.1. Expression and Purification of FtsQ_p_

His_6_-Avi-TEV-FtsQ_p_ was expressed from BL21(λDE3) *pET16b-His-avi-TEV-FtsQ_p_* and purified as previously reported [13]. The protein has a hexahistidine-tag and an Avi-tag at the N-terminus for purification and enzymatic biotinylation, respectively. The sequence of the construct is MGHHHHHHGL NDIFEAQKIE WHEENLYFQG EDAQRLPLSK LVLTGERHYT RNDDIRQSIL ALGEPGTFMT QDVNIIQTQI EQRLPWIKQV SVRKQWPDEL KIHLVEYVPI ARWNDQHMVD AEGNTFSVPP ERTSKQVLPM LYGPEGSANE VLQGYREMGQ MLAKDRFTLK EAAMTARRSW QLTLNNDIKL NLGRGDTMKR LARFVELYPV LQQQAQTDGK RISYVDLRYD SGAAVGWAPL PPEESTQQQN QAQAEQQ. A biotinylated sample of Akt-PH (Akt aa 1–123, 14.7 kDa) was kindly provided by ZoBio bv.

### 4.2. Immobilization of FtsQ_p_ for TINS

The purified FtsQ_p_ was enzymatically biotinylated using the BirA biotin-protein ligase standard reaction kit provided by Avidity, L.L.C (Aurora, USA). Actigel ALD resin (Sterogene, Carlsbad, CA, USA) was used as a 50% slurry. The resin was washed three times with H_2_O (10 mL) and incubated overnight with streptavidin (2.3 mg/mL in PBS). The resin was equally divided over two 2 mL reaction tubes. Residual unreacted aldehyde groups were deactivated by incubation with 100 mM d_11_-Tris and 10% coupling solution in PBS (20 min, RT). The resin (bed volume 600 µL) was incubated with FtsQ_p_ (103 µM in 50 mM Tris, 300 mM NaCl, 2 mM DTT, 600 µL) for 20 min at RT. The same procedure was repeated for immobilization of biotinylated Akt-PH. The presence of the proteins remaining in solution and bound to the resin was examined by SDS-PAGE analysis (data not shown). Quantification of immobilized protein was monitored by absorption of the supernatant at 280 nm before and after incubation (yield: FtsQ_p_ = 70%, Akt-PH = 100%). A final concentration of 72 µM of both FtsQ_p_ and Akt-PH was achieved. The FtsQ_p_-resin was washed with PBS twice to remove the excess, unbound protein.

### 4.3. FtsQ_p_ Activity Assay

To measure activity of immobilized FtsQ_p_, a pull down of the partner proteins FtsB_p_L_p_ was done. A solution of FtsB_p_L_p_ was added to a small aliquot of the FtsQ_p_-resin (FtsQ_p_:FtsB_p_L_p_ = 1:1) and incubated. The presence of FtsB_p_L_p_ in the supernatant and on the resin was analyzed before and after incubation by SDS-PAGE/Coomassie. The same experiment was done with an aliquot of FtsQ_p_-resin that was kept at 4 °C for 5 days. In both cases, FtsB_p_L_p_ was pulled down from the solution and present on the FtsQ_p_-resin, confirming that the immobilized protein was both functional and stable.

### 4.4. Target Immobilized NMR screening

Immobilized FtsQ_p_ and Akt-PH were each packed into a separate cell of a dual-cell sample holder [25]. Mixes of the 1501 fragments were made by 200-fold dilution in assay-buffer (20 mM NaP_i_ pH 7.5, 50 mM NaCl, 4% d_6_-DMSO in D_2_O) of a 100 mM d_6_-DMSO stock solution of each compound. Upon injection of each mix into the dual-cell sample holder, the flow was stopped and spatially selective Hadamard spectroscopy [27] was used to acquire a one-dimensional ^1^H-NMR spectrum of the contents of each cell separately. A background spectrum of only assay-buffer was recorded and subtracted from all TINS-spectra with compound mixes to remove the broad resonances from the sepharose resin. The cycle time was about 11 min, with 8 min required for the NMR experiment and 3 min for sample handling, resulting in a total time of about 4 days to complete the screen.

### 4.5. Production of ^15^N-Labeled FtsQ_p_

BL21 (λDE3) pLysS *pET16b-His-avi-TEV-FtsQ_p_* was expressed in minimal medium M9 (with ^15^NH_4_Cl as sole nitrogen source, supplemented with glucose, vitamins and trace elements) at 37 °C and 160 rpm. At an OD_600_ of 0.6, the culture was induced by adding IPTG to a concentration of 1 mM. After three hours of induction, the cells were harvested (6000× *g*, 15 min, 4 °C). Labeled FtsQ_p_ (^15^N-FtsQ_p_) was purified from the lysate by affinity chromatography using a TALON column (GE Healthcare) as described in our previous work [13] at a yield of 8 mg protein per liter culture. The N-terminal tags were cleaved off by incubation with His-tagged TEV protease (4 °C, o/n), cleaving the protein at ENLYFQ\G (see Section 4.1). The cleaved-off tags and the protease were removed from the protein by affinity chromatography using a TALON column (GE Healthcare), yielding the ^15^N-labeled FtsQ_p_ protein sample.

### 4.6. Biosensor Analysis

A BIAcore T200 SPR-based biosensor instrument (GE Healthcare, Uppsala, Sweden) was used in all experiments. NeutrAvidin (Fisher) was coupled to the surface of the active and reference channel of a Series S CM5 sensor chip (GE Healthcare) using the BIAcore amine coupling protocol [31]. Immobilization and interaction studies were conducted at 25 °C in PBS with 0.005% P20 and 3% DMSO, using a contact time of 20 s, a dissociation time of 40 s and a flow rate of 60 µl/min. Enzymatically biotinylated FtsQ_p_ was captured on the NeutrAvidin surface in the active channel in a manual run. In this case, considering the fragments having roughly a 100 times smaller molecular weight than the protein, we immobilized 10,000 response units of FtsQ_p_ on the chip, expecting a maximum response of the fragments of approximately 100 RUs. In the reference channel, chemically biotinylated Akt-PH was captured on the NeutrAvidin surface.

### 4.7. Hit Validation by ^1^H,^15^N-HSQC NMR

Twenty selected hits from the TINS screen were tested at 1 mM and 4 mM in ^15^N-FtsQ_p_, to look for chemical shift perturbations in the ^1^H,^15^N-HSQC spectrum. In all experiments, ^15^N-FtsQ_p_ was present at 200 µM concentration in a buffer containing 20 mM Na_2_HPO_4_ and 50 mM NaCl at pH 7.5. ^1^H,^15^N-HSQC experiments were acquired at 25 °C in a Bruker AV 600 MHz spectrometer equipped with a cryoprobe. During the experiment, the proper folding of the protein was confirmed by repeatedly injecting one of the fragment mixtures as a control. A reference titration of DMSO was used to subtract chemical shift perturbations not related to fragment binding.

## 5. Conclusions

In this work, we have explored the ligandability of FtsQ with the ultimate aim to find novel ligands for the periplasmic domain of FtsQ. We consciously chose to use a fragment-based approach, using biophysical techniques that are able to identify low affinity ligands. From a library of 1501 fragments, we were able to identify only three weak-affinity hits (KD > 1 mM) suggesting that the ligandability of FtsQ is low. This indicates that it is not trivial to develop high affinity ligands for FtsQ. An alternative approach would be to screen larger compounds or even small peptides, but this will expectedly bring up other challenges, since compounds larger than 500–600 Da are likely unable to cross the Gram-negative outer membrane. The hit fragments described in this study are the first small molecule ligands for FtsQ that have been identified. These ligands can be used as tool compounds in other approaches searching for inhibitors of FtsQ. Further investigation, such as assignment of the NMR spectrum of FtsQ, will be necessary to confirm the binding sites of these fragments. Such studies can also offer insights on how to optimize the fragments into more potent inhibitors of the essential interactions of FtsQ. In addition, ligands of FtsQ may influence its recently described role in the inhibition of septal peptidoglycan synthesis [32].

## Abbreviations

PPI	protein-protein interaction
NMR	nuclear magnetic resonance
TINS	target immobilized NMR screening
SPR	surface plasmon resonance
HSQC	heteronuclear single quantum coherence
CSP	chemical shift perturbation
FBDD	fragment-based drug discovery
TPSA	total polar surface area
HTS	high throughput screening
T/R	target spectrum/reference spectrum ratio
